# Supplementary material for: SimiC enables the inference of complex gene regulatory dynamics across cell phenotypes
Source: Commun Biol. 2022 Apr 12;5:351. doi: 10.1038/s42003-022-03319-7 (PMC9005655; doi:10.1038/s42003-022-03319-7)
Supplement: Supplementary file 2 — Supplementary Information [file 42003_2022_3319_MOESM2_ESM.pdf]

Supplementary Material for “SimiC enables the inference of complex gene regulatory dynamics across cell phenotypes.”

Jianhao Peng, Guillermo Serrano, Ian M. Traniello, Maria E. Calleja-Cervantes, Ullas V. Chembazhi, Sushant Bangru, Auinash Kalsotra, Teresa Ezponda, Juan Roberto Rodriguez-Madoz, Felipe Prosper, Idoia Ochoa*, Mikel Hernaez*

Corresponding authors. Email: idoia@illinois.edu, mhernaez@unav.es

Contents

S 1 Supplementary Tables **3**

S 2 Supplementary Figures **7**

S 3 Supplementary Note 1 **16**

S 3.1 Commands used to obtain the results for SCENIC 16
S 3.2 Commands used to obtain the results for SINCERITIES 17
S 3.3 Commands used to obtain the results for ICAnet 18
S 3.4 Commands used to obtain the results for SimiC 20

List of Tables

Supplementary Table 1	Results of the ChiP-Seq evidence for the monocyte dataset	4
Supplementary Table 2	Results of the ChiP-Seq evidence for the CD4+ T-lymphocytes dataset	5
Supplementary Table 3	Description of the datasets	6

List of Figures

Supplementary Figure 1	Cohen's kappa coefficient obtained on the synthetic data with SimiC, c.LASSO and SINCERITIES for different thresholds.	7
Supplementary Figure 2	Goodness of fit (measured by the adjusted R^2) and number of transcription factors regulating the target genes for the analyzed real datasets.	8
Supplementary Figure 3	Additional results regarding assigned weights and activity scores of several regulons inferred by SimiC for various datasets	9
Supplementary Figure 4	Analysis of the regulons and modules inferred by SCENIC and ICAnet, respectively, on the CAR T cell dataset	10
Supplementary Figure 5	Modules inferred by ICAnet containing RUNX3 on the CAR T cells	11
Supplementary Figure 6	Analysis of all the scores computed by SCENIC, ICAnet and SimiC on the CAR T cell dataset	12
Supplementary Figure 7	Clustering results of SimiC, SCENIC and ICAnet on the CD4+ T-lymphocytes cells	13
Supplementary Figure 8	Additional results of SimiC for the hepatocyte dataset	14
Supplementary Figure 9	Evaluation of different imputation methods used as input to SimiC	15

S 1 Supplementary Tables

Transcription Factor	Odds ratio	Targets inferred by SimiC: With CHIP-Seq evidence	Targets inferred by SimiC: Without CHIP-Seq evidence	Transcription Factor	Odds ratio	Targets inferred by SimiC: With ChipSeq evidence	Targets inferred by SimiC: Without ChipSeq evidence
AHR	2.4209334	85	18	MEF2A	18.4331055	4	1
ATF3	7.4978883	26	2	MEF2C	5.9282092	37	11
ATF4	2.9630598	15	19	MXD4	1.1189897	3	5
BACH1	4.3775359	49	25	NFE2	5.4219379	164	30
BCL6	4.5304360	114	25	NFE2L2	0.9668978	4	6
BHLHE40	0.9505501	4	5	NFIL3	3.6513062	138	59
CEBPA	7.5685966	95	9	NFKBIA	1.2789404	3	261
CEBPB	3.8799309	154	17	NFKBIZ	3.3213372	144	87
CEBPD	6.1966466	213	61	NONO	9.2076705	57	15
CUX1	3.5059642	107	80	NR4A1	3.9580502	36	32
ELF1	6.2987322	268	42	POU2F2	4.6308920	319	52
ETV6	0.9902268	2	8	RBPJ	2.1634393	36	18
FLI1	4.3930636	90	15	RUNX1	Inf	24	0
FOS	3.5783291	192	35	RXRA	3.4971783	17	6
FOSL2	3.7329709	140	43	SFPQ	6.8906385	43	37
GTF3A	0.0000000	0	186	SOX4	2.9117559	16	14
HHEX	0.0000000	0	29	SPI1	5.2559537	437	32
HIF1A	5.3150490	99	12	STAT1	3.2276457	62	19
IKZF1	13.6800635	55	4	STAT2	6.4613131	13	38
IRF1	3.6048313	6	3	STAT3	3.6470812	8	1
JARID2	1.6326102	2	3	TCF25	2.4640241	1	4
JDP2	0.0000000	0	8	TFDP1	3.7066129	102	101
JUN	4.6938915	37	3	TRPS1	0.0000000	0	8
JUNB	3.4277315	348	131	USF2	4.0261124	10	4
JUND	3.5951367	113	21	XBP1	1.9454443	189	62
KLF10	3.7823874	118	85	YBX1	1.9573284	40	637
KLF13	1.3175562	3	9	YY1	8.6304982	64	4
KLF3	8.4404662	4	33	ZBTB7A	3.2321853	26	10
KLF4	2.9737157	19	9	ZBTB7B	0.0000000	0	1
KLF6	5.3682047	60	17	ZEB2	4.4777216	102	34
LYL1	4.2432598	30	73	ZNF467	2.7657130	78	80
MAFB	2.9669603	112	137	ZNF524	2.6631704	3	5
MAZ	2.3720738	55	16				

Supplementary Table 1: ChiP-Seq evidence for the monocyte dataset [Granja et al., 2019]. Results of the odds ratio test between each regulon and empirical TF-target binding evidence obtained from ChIP-Seq data, on the monocytes dataset.

Transcription Factor	Odds ratio	Targets inferred by SimiC: With CHIP-Seq evidence	Without CHIP-Seq evidence	Transcription Factor	Odds ratio	Targets inferred by SimiC: With ChipSeq evidence	Without ChipSeq evidence
ARID4B	2.4690861	11	7	NCOR1	5.6515240	27	7
ARID5B	4.5884187	66	67	NFKB1	0.6646428	2	5
ATRX	2.6881720	1	18	NFKB2	0.0000000	0	1
BATF	5.5085389	65	27	NFKBIA	0.0000000	0	147
BCL11B	13.5380037	76	12	NFKBIZ	15.9520334	8	1
BHLHE40	Inf	2	0	NONO	4.8289855	4	2
CEBPB	Inf	8	0	NR3C1	2.6184494	16	3
CREM	8.2261561	6	1	PBX2	Inf	1	0
ELF1	8.4172264	43	5	POU2F2	Inf	9	0
ETS1	7.6077545	122	19	PRDM1	5.0717411	8	1
FLI1	Inf	2	0	RUNX2	0.9345519	7	7
FOS	3.1939239	108	22	RUNX3	4.8112994	103	39
FOSL2	1.7113995	6	4	SATB1	3.2277012	26	199
FOXP1	6.8588888	94	13	SFPQ	3.1681276	38	71
GATA3	5.9537226	69	8	SMAD3	Inf	1	0
GTF3A	0.0000000	0	109	SP140	Inf	1	0
ID3	0.0000000	0	1	STAT1	9.3820055	19	2
IKZF1	4.9706751	40	8	STAT3	11.8606038	26	1
IRF1	5.3105098	123	42	TCF25	2.3480087	5	21
JUN	2.9685132	39	5	TCF7	3.9431971	170	55
JUNB	3.6435228	40	14	THAP11	0.0000000	0	1
JUND	6.6351428	129	13	TSC22D4	2.5602017	15	113
KLF13	6.5932959	10	6	UBTF	3.3198623	4	3
KLF3	3.3015387	1	21	XBP1	2.0203395	73	23
KLF6	4.6004665	252	84	YBX1	1.8544620	31	519
LEF1	3.6710235	90	165	YY1	0.8075069	3	2
MAF	2.9986765	15	3	ZBTB20	Inf	1	0
MAX	Inf	4	0	ZBTB7A	3.2324393	26	10
MAZ	4.8788522	211	30	ZNF331	0.0000000	0	27
MXD4	4.0401001	174	81	ZNF394	Inf	1	0
MXI1	3.1115985	27	13	ZNF75A	0.0000000	0	1
MYC	3.4385698	98	7				

Supplementary Table 2: ChiP-Seq evidence for the CD4+ T-lymphocytes dataset [Granja et al.**, 2019].** Results of the odds ratio test between each regulon and empirical TF-target binding evidence obtained from ChIP-Seq data, on the CD4+ T-lymphocytes dataset.

Dataset	Number of cells	Number of cell-types	Number of phenotypes	% sparsity (before imputation)	% sparsity (after imputation)	Reference
Synthetic	5,000	None	5	2.24	-	None
Bone marrow and peripheral blood (Monocytes)	6,291	2	2	88.71	0.0013	Granja et al., 2019
Bone marrow and peripheral blood (T-lymphocytes)	12,770	6	2	92.13	0.0047	Granja et al., 2019
Non-Hodgkin lymphoma CD4+ T-lymphocytes cells	16,194	3	2	88.61	18.69	Sheih et al., 2020
Liver regeneration	12,548	4	4	90.65	0.0003	Chembazhi et al., 2021
Honey bee brain	1,641	11	2	81.35	0	Traniello et al., 2020

Supplementary Table 3: Description of the datasets used in the study.

S 2 Supplementary Figures

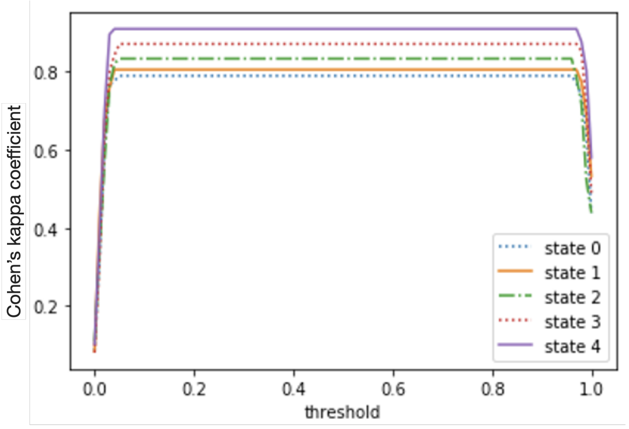

(A) SimiC

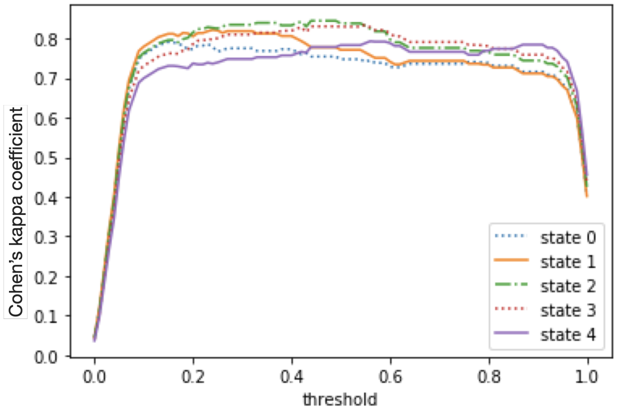

(B) c.LASSO

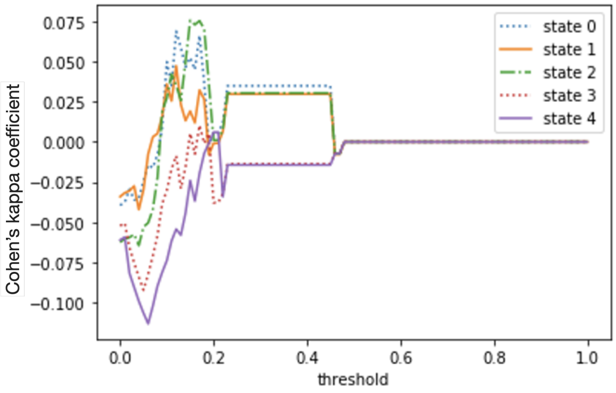

(C) SINCERITIES

Supplementary Figure 1: Cohen's kappa coefficient for the 5 states of the generated synthetic dataset obtained with **A:** SimiC, **B:** LASSO run in all states combined (denoted as c.LASSO) and **C:** SINCERITIES, for different thresholds. The threshold is used to convert the weight of the inferred edges into $0, +1$ and -1. Specifically, for a threshold t, all weights above t are converted to $+1$, all weights below $-t$ to -1, and the rest to 0. We observed that whereas the Cohen's kappa coefficient obtained with SimiC and c.LASSO is stable with the used threshold, that is not the case for SINCERITIES.

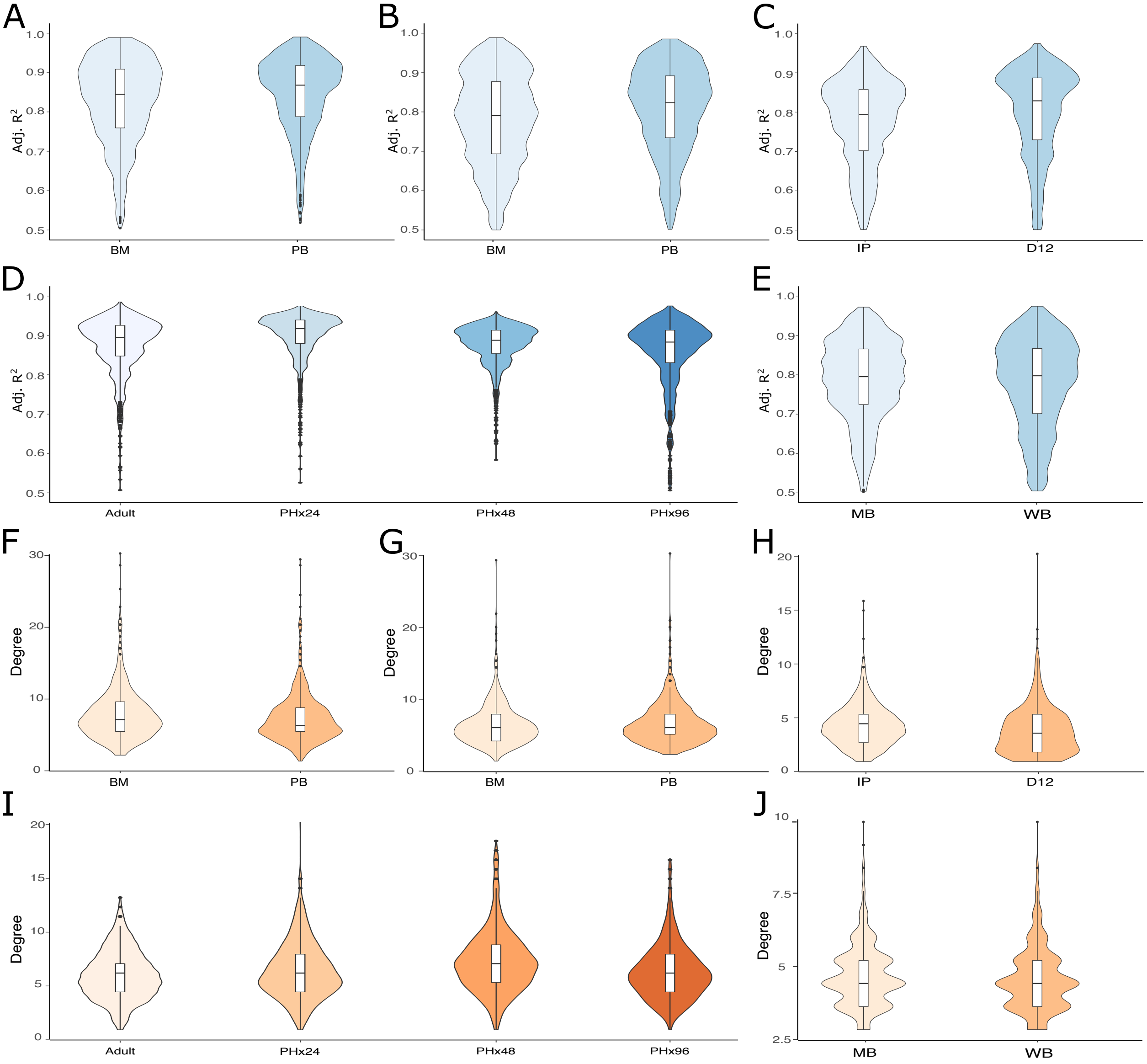

Supplementary Figure 2: Goodness of fit (measured by the adjusted R^2) and number of transcription factors regulating the target genes for all the analyzed real datasets. Violin plots (colored in blue) showing the distribution of the adjusted R^2 per target gene across the phenotypes on **A:** monocytes coming from either bone marrow (BM) or peripherial blood (PB) [Granja et al., 2019]; **B:** CD4+ T-lymphocytes coming from either BM or PB [Granja et al., 2019]; **C:** CAR T cells isolated from the infusion product (IP) as well as from PB at the expansion peak after treatment (D12) [Sheih et al., 2020]; **D:** hepatocytes sequenced at different timepoints (adult, PHx24, PHx48 and PHx96) after partial hepatectomy [Chembazhi et al., 2021]; **E:** bee brain cells coming from either WB or MB [Traniello et al., 2020]. The adjusted R^2 measures the goodness of fit between the true target genes' expressions and the ones computes with SimiC's inferred GRNs on the test data (which corresponds to about 20% of the data). Violin plots (colored in orange) showing the distribution of the number of transcription factors (TFs) regulating each target gene across the phenotypes on **F:** monocytes coming from either BM or PB [Granja et al., 2019]; **G:** CD4+ T-lymphocytes coming from either BM or PB [Granja et al., 2019]; **H:** CAR T cells at IP and D12 [Sheih et al., 2020]; **I:** hepatocytes sequenced at timepoints adult, PHx24, PHx48 and PHx96 [Chembazhi et al., 2021]; **J:** bee brain cells coming from either WB or MB [Traniello et al., 2020]. We observed that the median adjusted R^2 was above 0.8 in all cases, and the average number of transcription factors (TFs) regulating a given target gene was generally below 10.

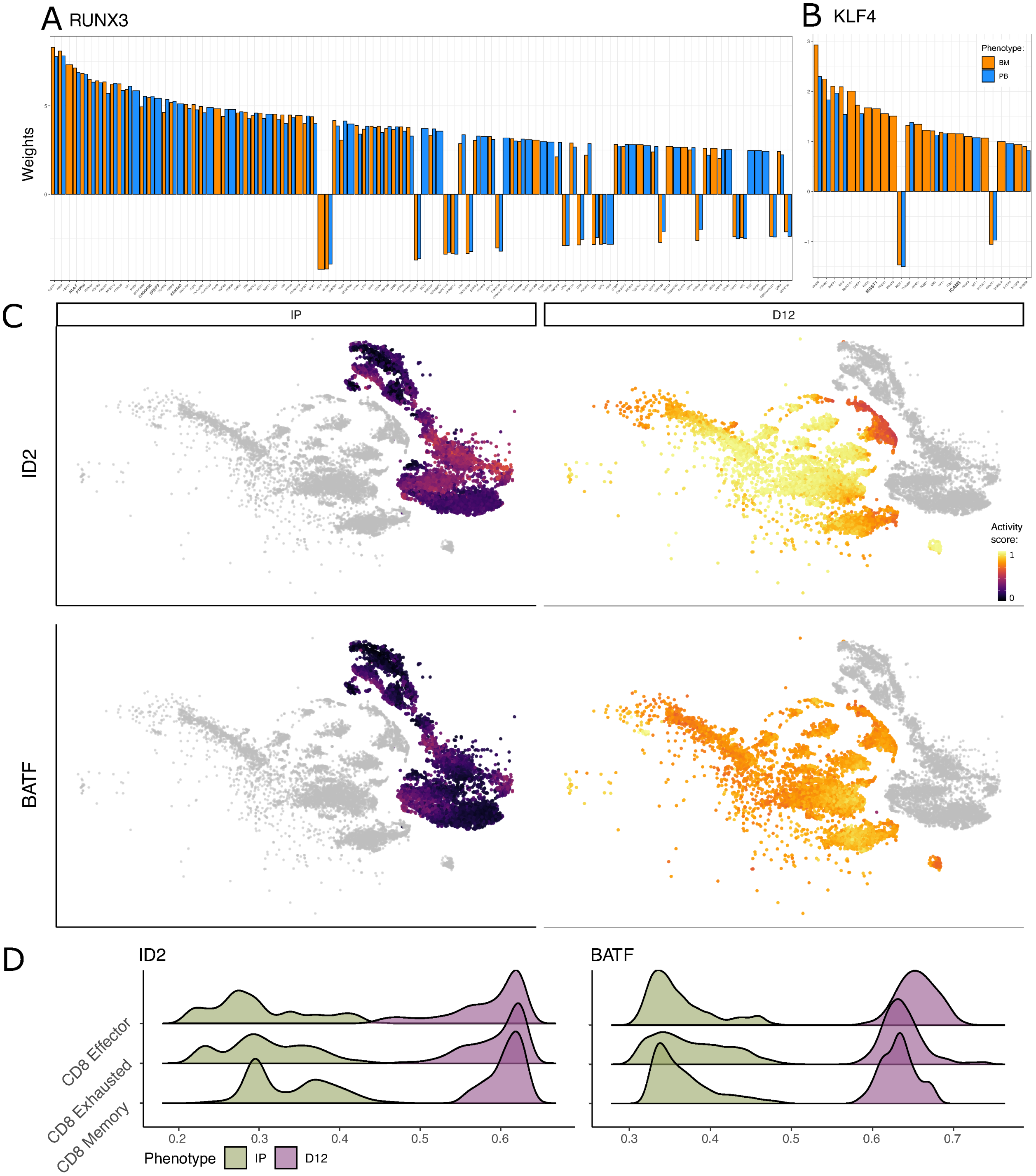

Supplementary Figure 3: Additional plots regarding the assigned weights of the KLF4 and RUNX3 regulons from monocyte and CD4+ T-lymphocytes, respectively, as well as the activity scores of the regulons ID2 and BATF across the different CAR T cells. Barplot showing the assigned weights to the target genes on the **A:** KLF4 regulon from monocytes coming from BM or PB [Granja et al., 2019] and **B:** on the RUNX3 regulon from the CD4+ T-lymphocytes coming also from either BM or PM [Granja et al., 2019]. **C:** tSNE depicting the activity of the regulons ID2 and BATF across the different cells on the two different phenotypes (IP and D12) of the CAR T cells [Sheih et al., 2020] and **D**: ridges showing their activity scores across the different cell types. These regulons show higher activity at D12 as compared to IP.

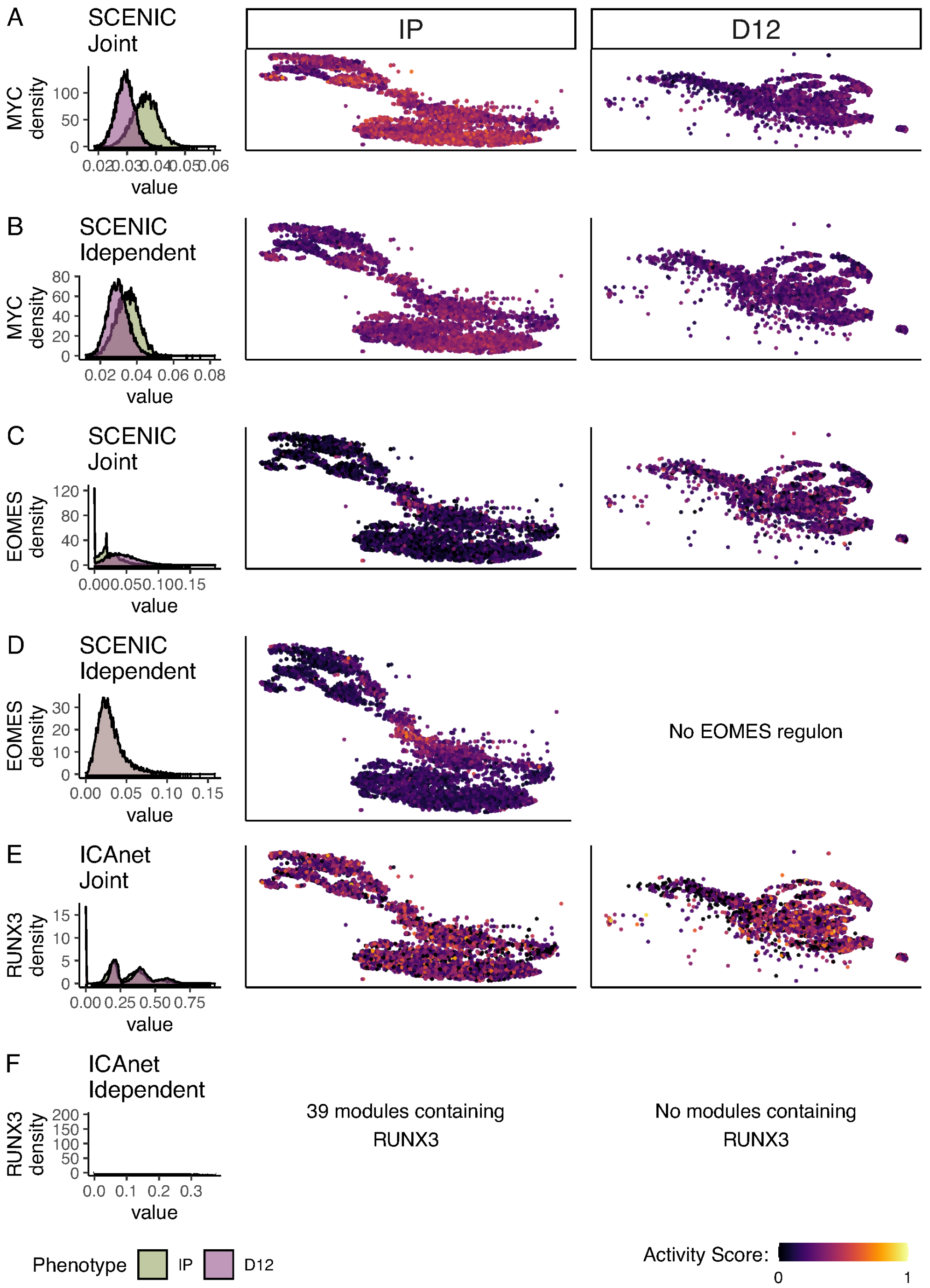

Supplementary Figure 4: Analysis of the MYC, EOMES, and RUNX3 regulons inferred by SCENIC and of the modules inferred by ICAnet containing those TFs on the CAR T cell dataset with phenotypes IP and D12 [Sheih et al., 2020]. A-B: Densities of the AUC score of the MYC regulon for the two phenotypes IP and D12, and tSNE visualization of the CAR T cells colored by the AUC score calculated jointly **(A)** and separately **(B)** with SCENIC. **C-D:** Densities of the AUC score of the EOMES regulon for the two phenotypes IP and D12, and tSNE visualization of the CAR T cells colored by the AUC score calculated jointly **(C)** and separately **(D)** with SCENIC. **E-F:** Densities of the score of the modules containing the RUNX3 TF for the two phenotypes IP and D12, and tSNE visualization of the CAR T cells colored by the module score calculated jointly **(E)** and separately **(F)** with ICAnet. The visualization of the 39 modules containing RUNX3 when ICAnet is run jointly are shown in Supplementary Figure Supplementary Figure 5.

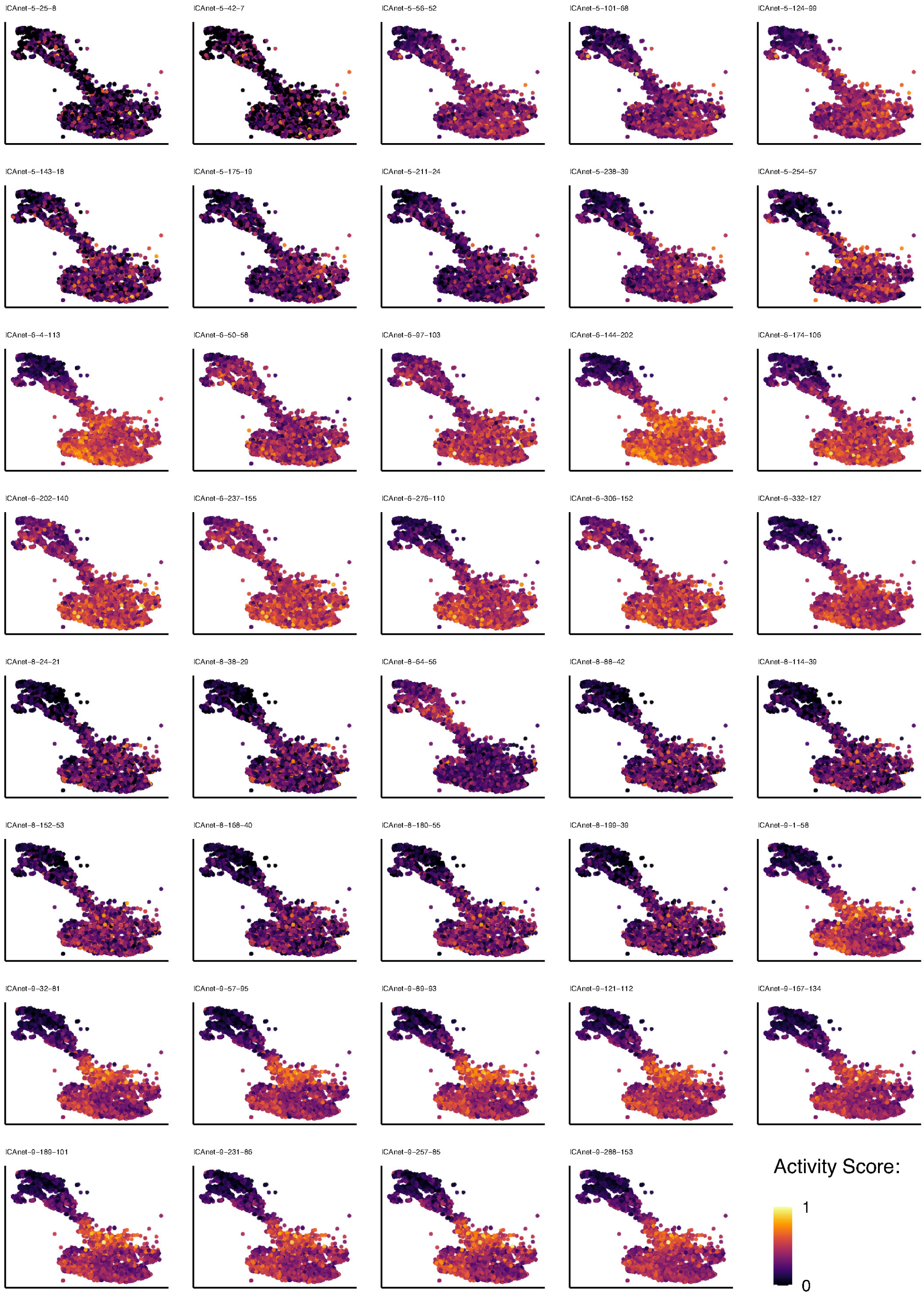

Supplementary Figure 5: Modules inferred by ICAnet containing RUNX3 on the IP phenotype of the CAR T cell dataset [Sheih et al.**, 2020]**. tSNE visualization of the scores produced by ICAnet, when run independently on the IP phenotype, on the 39 modules containing the RUNX3 transcription factor.

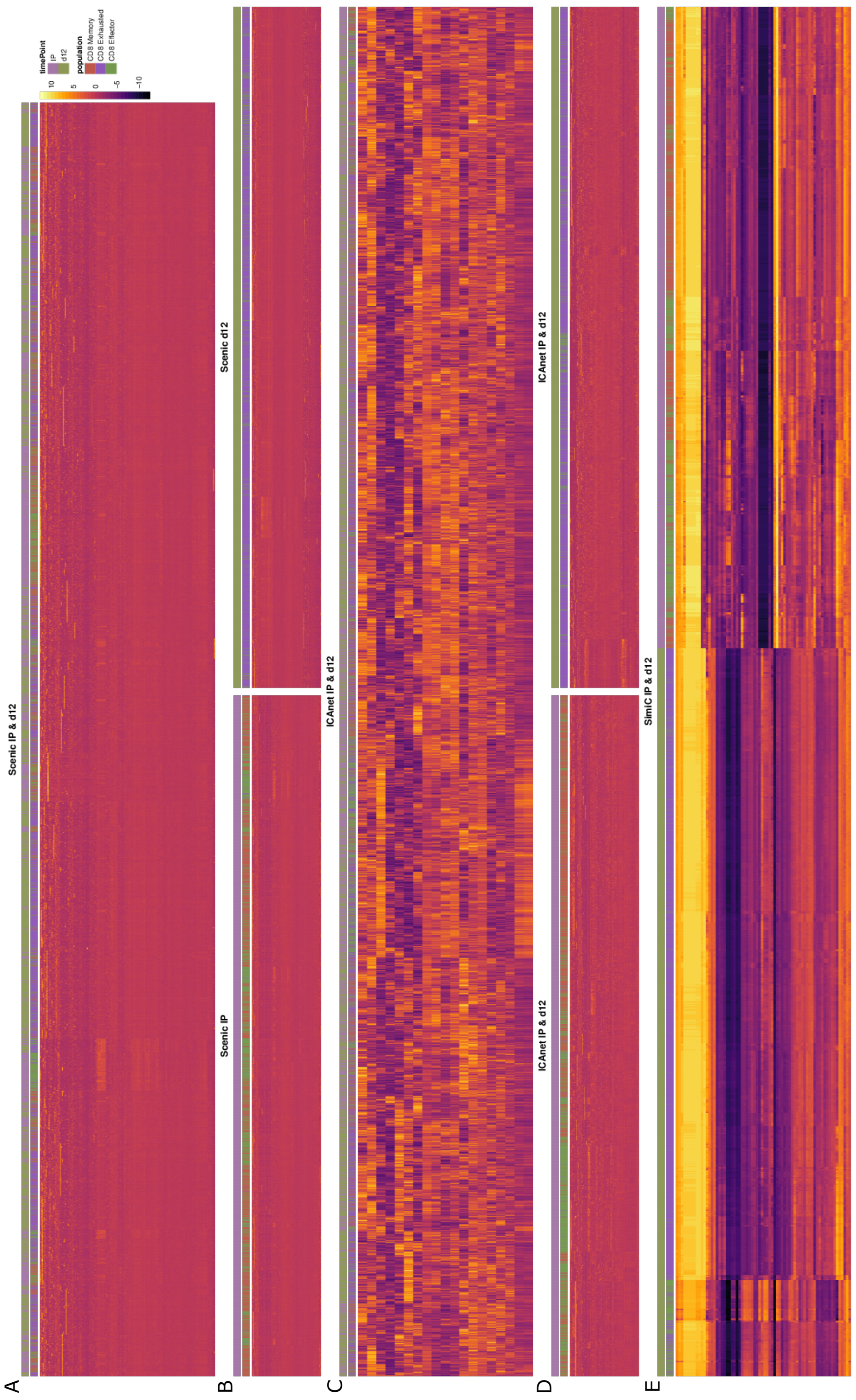

Supplementary Figure 6: Analysis of the scores computed by SCENIC, ICAnet and SimiC (for all inferred regulons or modules) on the CAR T cell dataset [Sheih et al., 2020]. We applied hierarchical clustering with Euclidean distance to the scores produced by each method, to analyze their capability to capture the phenotype (IP and D12) or cell state (CD8 Memory, CD8 Exhausted, and CD8 Effector) of the CAR T cells. **A-B:** Resulting heatmaps of the AUC scores computed by SCENIC for all the inferred regulons across all the cells when run jointly (**A**) and separately (**B**) on each phenotype. **C-D:** Resulting heatmaps of the module scores computed by ICAnet for all the inferred modules across all the cells when run jointly (**C**) and separately (**D**) on each phenotype. **E:** Heatmap of the activity score for all the regulons inferred by SimiC for both phenotypes across all the cells.

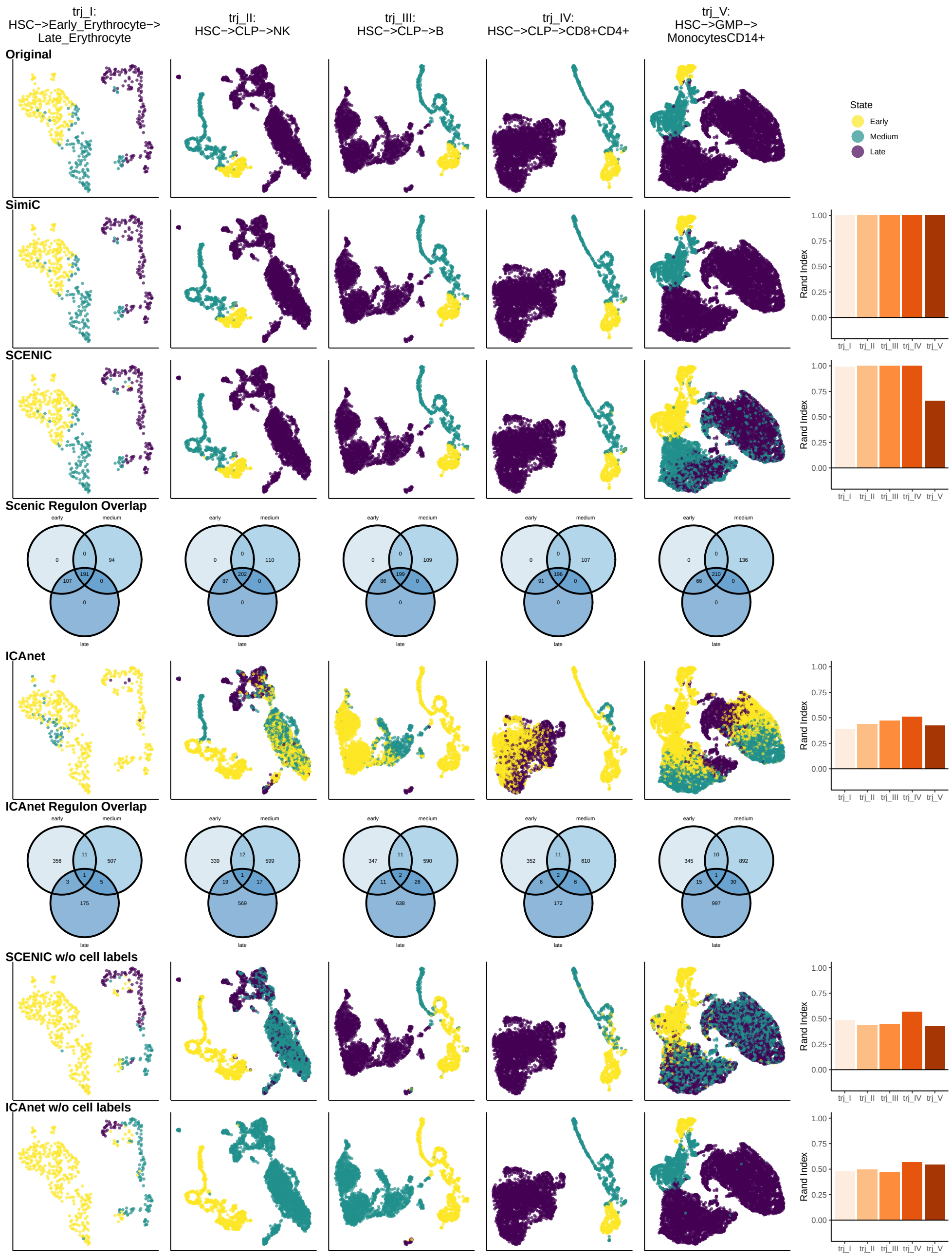

Supplementary Figure 7: Clustering results on the CD4+ T-lymphocytes cells [Granja et. al., 2019]. We selected five known differentiation trajectories, namely: i) HSC -> Early Erythrocyte -> Late Erythrocyte, ii) HSC -> CLP -> NK, iii) HSC ->CLP -> B, iv) HSC -> CLP -> CD8+ CD4+, and v) HSC -> GMP -> Monocytes CD14+. For ease of notation, we refer to the states of a given trajectory as Early -> Medium -> Late. The Umap plot for the original data shows the cells colored by their state (yellow, green, and purple, respectively), for each trajectory. We applied SimiC to the cells of each trajectory (using the corresponding ordering), and then clustered the cells with k-means ($k = 3$) using the generated regulon activity scores. The Umap plots and the corresponding Rand Index (RI) scores (close to 1 in all cases) demonstrate that SimiC's computed scores can reliably differentiate cells. We did a similar analysis with SCENIC and ICAnet, when run independently on each state and on the whole data (denoted as *w/o cell labels*). In the former case, we also show the overlap in the generated regulons (for SCENIC) or modules (for ICAnet) across the three states. For clustering, we used k-means for SCENIC and Seurat for ICAnet. As can be observed, when run independently on each state, SCENIC obtains high RI scores for all trajectories except the last one, where the score drops below 0.75. When no states are given to SCENIC, the scores drop significantly (most below 0.5). ICAnet fails to provide scores that can cluster cells by their state, in both considered cases.

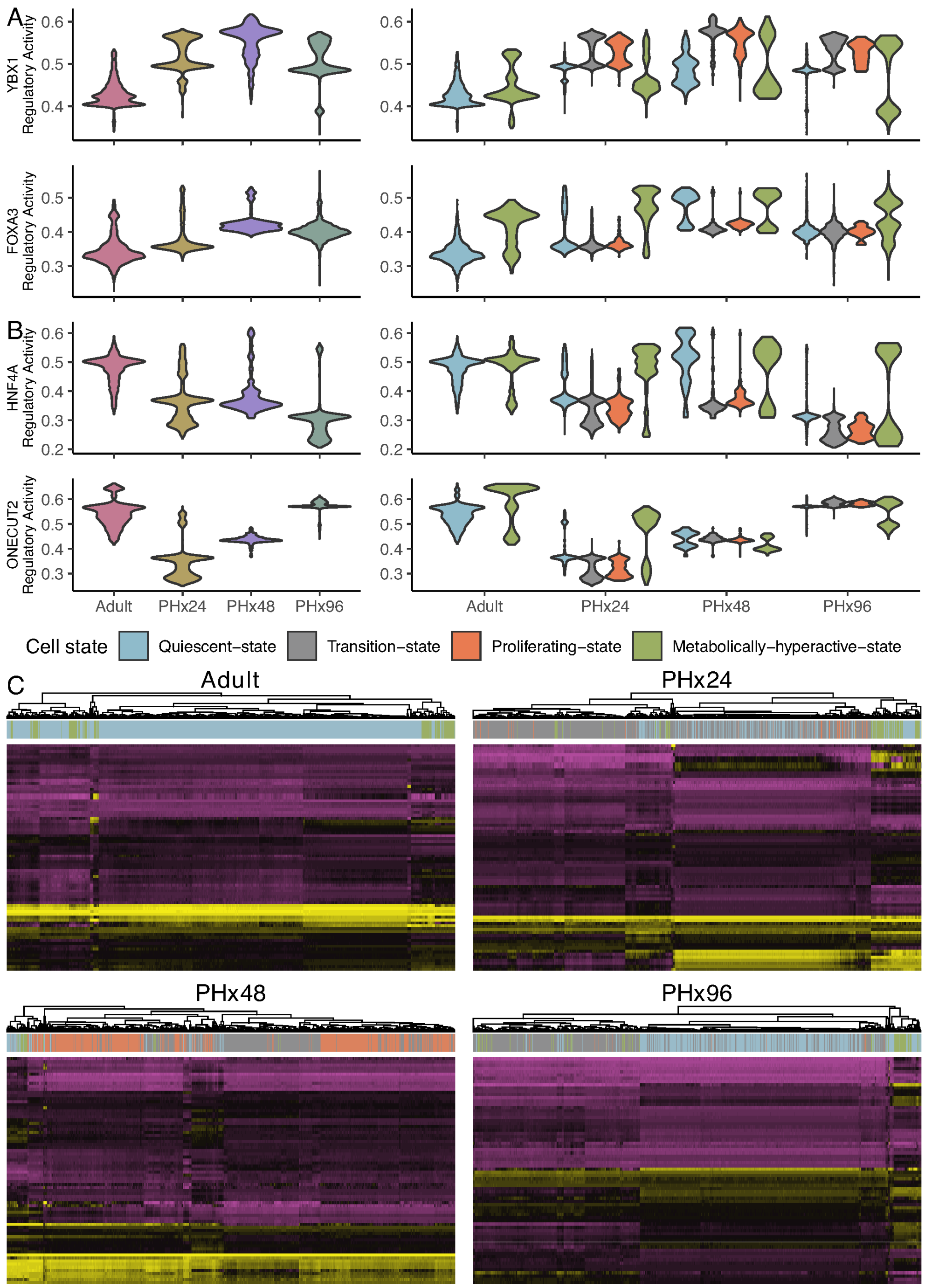

Supplementary Figure 8: Additional results of SimiC for the hepatocyte dataset at timepoints adult, PHx24, PHx48 and PHx96 of a regenerating liver [Chembazhi et al., 2021]. A: Violin plots showing the activity score distribution of the YBX1 (top) and FOXA3 (bottom) regulons for each timepoint, demonstrating their initiation-progression pattern. The activity score is also split by cell state. **B:** Violin plots showing the activity score distribution of the HNF4A (top) and ONECUT2 (bottom) regulons for each timepoint, demonstrating their termination-rematuration pattern. The activity score is also split by cell state. **C:** Heatmaps of the regulon activity scores for each cell, for each of the timepoints. We also show the results of applying hierarchical clustering with Euclidean distance. Note that cells cluster accurately by cell state (quiescent, transition, proliferating and metabolically-hyperactive) for all the different timepoints, showing that SimiC's regulon activity score is able to capture cell-state regulon activities across the different liver regenerating stages.

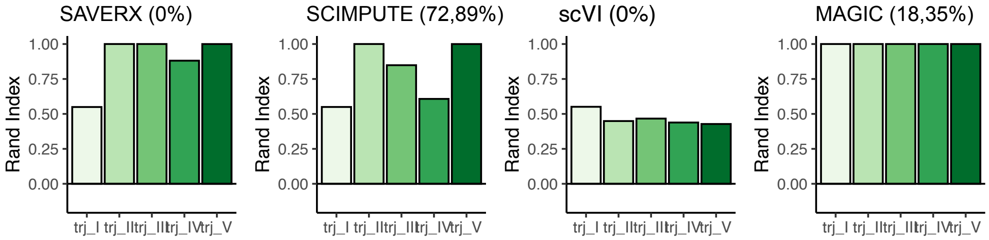

Supplementary Figure 9: Evaluation of different imputation methods used as input to SimiC. Rand Index (RI) scores assessing the clustering accuracy on SimiC's computed scores for each trajectory defined on Supplementary Figure Supplementary Figure 7, for imputation methods SAVERX [Wang et al., 2018], scImpute [Li and Li, 2018], scVI [Lopez et al., 2018], and MAGIC [Van Dijk et al., 2018]. For each method, the sparsity of the resulting imputed dataset is specified in parenthesis by the side of their name. For reference, the sparsity of the original data (CD4+ T-lymphocytes cells [Granja et. al., 2019]) used on the trajectories is 91,11%.

S 3 Supplementary Note 1

S 3.1 Commands used to obtain the results for SCENIC

```
import scanpy as sc
import numpy as np
import loompy as lp

#Load the data
adata = sc.read_csv(cart_data_path, delimiter = '\t',
                    first_column_names=True)

# compute the number of genes per cell (computes 'n_genes' column)
sc.pp.filter_cells(adata, min_genes=0)

# mito and genes/counts cuts
mito_genes = adata.var_names.str.startswith('MT-')

# for each cell compute fraction of counts in mito genes vs. all genes
adata.obs['percent_mito'] = np.ravel(np.sum(
    adata[:, mito_genes].X, axis=1)) / np.ravel(np.sum(adata.X,
    axis=1))

# add the total counts per cell as observations-annotation to adata
adata.obs['n_counts'] = np.ravel(adata.X.sum(axis=1))

sc.pp.filter_cells(adata, min_genes=200)
sc.pp.filter_genes(adata, min_cells=3)
adata = adata[adata.obs['n_genes'] < 4000, :]
adata = adata[adata.obs['percent_mito'] < 0.15, :]

row_attrs = {
"Gene": np.array(adata.var_names),
}
col_attrs = {
"CellID": np.array(adata.obs_names),
"nGene": np.array(np.sum(adata.X.transpose()>0, axis=0)).flatten(),
"nUMI": np.array(np.sum(adata.X.transpose(),axis=0)).flatten(),
}

lp.create("cart_filtered.loom",adata.X.transpose(),row_attrs,
col_attrs)
# End of the Python commands, the rest should be executed on the terminal.

# generate the GRN
! pyscenic grn --num_workers 20 --output adj.tsv \
--method grnboost2 cart_filtered.loom  hs_hgnc_tfs.txt

# calculate the possible regulons
! pyscenic ctx adj.tsv  hg38__refseq-r80__10kb_up_and_down_tss.mc9nr.feather \
--annotations_fname motifs-v9-nr.hgnc-m0.001-o0.0.tbl \
--expression_mtx_fname cart_filtered.loom  --mode "dask_multiprocessing" \
--output reg.csv  --num_workers 20  --mask_dropouts

# calculate the cellular enrichment
! pyscenic aucell  PBMC10k_filtered.loom  reg.csv \
--output cart_SCENIC.loom  --num_workers 20
```

S 3.2 Commands used to obtain the results for SINCERITIES

```
###################
# PACKAGES required:
# glmnet
# ppcor
# cvTools
###################
library(glmnet)
library(ppcor)
library(cvTools)

# *** Data loading ***
uploading <- dget("SINCERITIES functions/uploading.R")
# DATA <- uploading('THP1 data/THP1_single_cell_data_EXCEL_no6_24_72_96.csv')
DATA <- uploading('synthetic_d50t20_5000_cells_w_header.csv')

# *** SINCERITIES ***

SINCERITIES_PLUS <- dget("SINCERITIES functions/SINCERITIES_PLUS.R")
result <- SINCERITIES_PLUS(DATA,noDIAG = 0,SIGN = 1,CV_nfolds = 10)
adj_matrix <- result$adj_matrix
SIGN <- 1

# Final ranked list
adj_matrix <- adj_matrix/max(adj_matrix)
final_ranked_predictions <- dget("SINCERITIES functions/final_ranked_predictions.R")
table <- final_ranked_predictions(adj_matrix,DATA$genes,SIGN=1,
directory = "Results", fileNAME='synthetic_d50t20_simic_cmp',saveFile = TRUE)
```

S 3.3 Commands used to obtain the results for ICAnet

```
library(doParallel)
library(Seurat)
library(SingleCellExperiment)
library(cowplot)
library(ica)
library(ICAnet)
library(RcisTarget)
library(mclust)
library(RSCORE)

load('./Data/clonalKinetics_seuratobj_noCARexpres.Robj')
load('./Data/clonalKinetics_subseted_noCARexpres.Robj')
cart_data <- seuratObj

Idents(cart_data) <- cart_data$TimePoint
cart_data <- subset(cart_data, idents=c('IP', 'd12'))

batch <- c(cart_data$TimePoint)
cluster <- c(subseted_object_forsimic$ClusterNames)
cart_data$batch <- batch
cart_data$cluster <- cluster


# integration
cart_data.list <- SplitObject(cart_data, split.by='TimePoint')
cart_data.list <- lapply(X = cart_data.list, FUN = function(x) {
    x <- NormalizeData(x)
    x <- FindVariableFeatures(x, selection.method = "vst", nfeatures = 2000)
})

features <- SelectIntegrationFeatures(object.list = cart_data.list, nfeatures=3000)
cart_data.all <- cart_data.list[[1]]
cart_data.all <- GetAssayData(cart_data.all)[features,]
cart_data.set <- cart_data.list[[2]]
cart_data.set <- GetAssayData(cart_data.set)[features,]
cart_data.all <- cbind(cart_data.all, cart_data.set[features,])

cart_data[['Consensus.RNA']] <- CreateAssayObject(cart_data.all)
DefaultAssay(cart_data) <- 'Consensus.RNA'

cart_data <- ScaleData(cart_data)
cart_data <- RunPCA(cart_data, npcs=50, verbose=FALSE, features=rownames(cart_data))
cart_data <- RunUMAP(cart_data, reduction='pca', dims=1:20, reduction.name='umap', reduction.key='umap_')

p1 <- DimPlot(cart_data, reduction='umap', group.by='TimePoint', label=1) +NoLegend()
p2 <- DimPlot(cart_data, reduction='umap', group.by='cluster', label=1)

pdf('./Plots/Integration_ica_cart.pdf')
plot_grid(p1,p2)
dev.off()


hs_network_matrix <- getPPI_String(cart_data, species=9606)
ica.cart_data <- ICAcomputing(cart_data, ICA.type = 'JADE', two.stage=FALSE, global.mode=FALSE, center=FALSE, scale=TRUE)

pdf('./Plots/heatmap_cart.pdf')
ica.filter <- CrossBatchGrouping(ica.cart_data$ica.pooling, cor='spearman', Unique.Preservation=FALSE)
dev.off()


cart_data <- RunICAnet(cart_data, ica.filter$ica.filter, PPI.net = hs_network_matrix, W.top=2, aucMaxRank=300, scale=TRUE)
cart_data <- RunModuleSVD(cart_data, nu=30, power=0.2)
cart_data <- RunUMAP(cart_data, reduction = 'Module_SVD', dims=1:20, reduction.name='umap', reduction.key = 'umap_', verbose=FALSE)

cart_data@active.ident <- as.factor(cart_data$cluster)
# beta here is the cell type
modules <- FindMarkerModule(cart_data, identity='2')
head(modules)

# library(networkD3)

# plot_module(gene_sets = cart_data@misc$IcaNet_geneSets[['ICAnet-2-3-3']], network=hs_network_matrix, nodeSize='ica.score', ica.score=abs(ica.filter$i
```

```
pdf('./Plots/test_Umap_CARTicascore.pdf')
FeaturePlot(cart_data, 'ICAnet-2-11-12', reduction='umap')
dev.off()

saveRDS(cart_data, './Data/ICA_results_cart.rds')
ica_activity <- as.data.frame(cart_data@assays$IcaNet@counts)






# Run the pipeline for Clustering
load('./Data/clonalKinetics_seuratobj_noCARexpres.Robj')
load('./Data/clonalKinetics_subseted_noCARexpres.Robj')
cart_data_clust <- seuratObj

Idents(cart_data_clust) <- cart_data_clust$TimePoint
cart_data_clust <- subset(cart_data_clust, idents=c('IP', 'd12'))

batch <- c(cart_data_clust$TimePoint)
cluster <- c(subseted_object_forsimic$ClusterNames)
cart_data_clust$batch <- batch
cart_data_clust$cluster <- cluster


# integration
seurat_int_data.list <- SplitObject(cart_data_clust, split.by = "TimePoint")
seurat_int_data.list <- lapply(X = seurat_int_data.list, FUN = function(x) {
    x <- NormalizeData(x)
    x <- FindVariableFeatures(x, selection.method = "vst", nfeatures = 2000)
})
# select features that are repeatedly variable across datasets for integration
features <- SelectIntegrationFeatures(object.list = seurat_int_data.list)
seurat_int_data.anchors <- FindIntegrationAnchors(object.list = seurat_int_data.list, anchor.features = features)
seurat_int_data.combined <- IntegrateData(anchorset = seurat_int_data.anchors)

DefaultAssay(seurat_int_data.combined) <- "integrated"
seurat_int_data.combined  <- ScaleData(seurat_int_data.combined )
cart_data_clust <- NormalizeData(cart_data_clust, normalization.method='LogNormalize', scale.factor=10000)

Motif_Net <- TF_Net_Generate('./Data/hg19-500bp-upstream-7species.mc8nr.feather')
load('./Data/motifAnnotations_hgnc_v8.rdata')

ica.cart_data_clust <- ICAcomputing(cart_data_clust, ICA.type='JADE', RMT=TRUE, two.stage=FALSE)
cart_data <- RunICAnetTF(cart_data_clust, ica.cart_data_clust$ica.pooling, W.top.TFs=2, W.top.genes=1.5, aucMaxRank=600, Motif_Net=Motif_Net, TF_motif_
moduleInfor <- cart_data@misc$IcaNet_geneSets_TF_moduleInfor

cart_data <- RunPCA(cart_data, npcs =30, features=rownames(cart_data), verbose=FALSE)
pdf('./Plots/elbow_clusters_cart_data.pdf')
ElbowPlot(cart_data, ndims=30)
dev.off()


cart_data <- FindNeighbors(cart_data, dims=1:20, reduction='pca')
cart_data$seurat_clusters <- NULL
cart_data <- FindClusters(cart_data, resolution= 0.1, algorithm=2)

saveRDS(cart_data ,  './Data/ICA_results_cart_cluster_END.rds')
```

S 3.4 Commands used to obtain the results for SimiC

```
from simiclasso.clus_regression import simicLASSO_op
from simiclasso.weighted_AUC_mat import main_fn


p2df = 'ClonalKinetic.DF.pickle' # File containing the scRNA data
p2assignment = ClonalKinetics.clustAssign.txt' # File containing the cell phenotype, in this case, IP or D12
p2tf = 'ClonalKinetics.TFs.pickle' # File containing the list of the TFs to use for the regulon inference

similarity = True #Confirm the use of constrains
k_cluster = None #Do not use any clustering information from the scRNA dataset
num_TFs = -1    #Use all the TFs present on the TFs file
num_target_genes = -1 #Use all the targets to compute the regulons
max_rcd_iter = 100000
df_with_label = False

percent_of_target = 1 #Use all the targets to compute the regulatory activity
cross_val = True
p2saved_file_cross = 'ClonalKinetics_CrossVal_Ws.pickle'

# perform the cross-validation in order to select the optimal Lambda1 and Lambda2
simicLASSO_op(p2df, p2assignment, similarity, p2tf, p2saved_file_cross, \
              k_cluster, num_TFs, num_target_genes, max_rcd_iter = max_rcd_iter,\
              df_with_label = df_with_label, cross_val=cross_val)

#Once selected the optimal Lambda1 and Lambda2, fixe them and calculate the weights
cross_val = False
lambda1= 0.01
lambda2 = 0.01
p2saved_file = 'ClonalKinetics_L1'+str(lambda1)+'_L2'+str(lambda2)+'_Ws.pickle' #Weigths
p2AUC =        'ClonalKinetics_L1'+str(lambda1)+'_L2'+str(lambda2)+'_AUCs.pickle' #Auc

simicLASSO_op(p2df, p2assignment, similarity, p2tf, p2saved_file, \
              k_cluster, num_TFs, num_target_genes, max_rcd_iter = max_rcd_iter,\
              df_with_label = df_with_label, lambda1=lambda1, lambda2 = lambda2)

#Once the weights have been calculated, we can compute the regulatory activity for each regulon
main_fn(p2df, p2saved_file, p2AUC, percent_of_target = percent_of_target)
```

Supplementary References

[Chembazhi et al., 2021] Chembazhi, U. V., Bangru, S., Hernaez, M. and Kalsotra, A. (2021). Cellular plasticity balances the metabolic and proliferation dynamics of a regenerating liver. Genome research .

[Granja et al., 2019] Granja, J. M., Klemm, S., McGinnis, L. M., Kathiria, A. S., Mezger, A., Corces, M. R., Parks, B., Gars, E., Liedtke, M., Zheng, G. X. et al. (2019). Single-cell multiomic analysis identifies regulatory programs in mixed-phenotype acute leukemia. Nature biotechnology *37*, 1458–1465.

[Sheih et al., 2020] Sheih, A., Voillet, V., Hanafi, L.-A., DeBerg, H. A., Yajima, M., Hawkins, R., Gersuk, V., Riddell, S. R., Maloney, D. G., Wohlfahrt, M. E. et al. (2020). Clonal kinetics and single-cell transcriptional profiling of CAR-T cells in patients undergoing CD19 CAR-T immunotherapy. Nature communications *11*, 1–13.

[Traniello et al., 2020] Traniello, I. M., Bukhari, S. A., Kevill, J., Ahmed, A. C., Hamilton, A. R., Naeger, N. L., Schroeder, D. C. and Robinson, G. E. (2020). Meta-analysis of honey bee neurogenomic response links Deformed wing virus type A to precocious behavioral maturation. Scientific Reports .
