# Supplementary material for: SimiC enables the inference of complex gene regulatory dynamics across cell phenotypes
Source: Commun Biol. 2022 Apr 12;5:351. doi: 10.1038/s42003-022-03319-7 (PMC9005655; doi:10.1038/s42003-022-03319-7)
Supplement: Supplementary file 3 — Description of Additional Supplementary Files [file 42003_2022_3319_MOESM3_ESM.pdf]

Description of Additional Supplementary Files

File name: Supplementary Data 1
Description: Results of the ChipSeq analysis in Supplementary Table S1 and S2

File name: Supplementary Data 2
Description: Gene markers used to annotate the different populations from the CAR-T dataset.

File name: Supplementary Data 3
Description: Results from the differential expression analysis between clusters that were used to annotate the different Apis mellifera brain cells.
